# Frequent video game playing alters low-frequency event-related EEG brain oscillations

**DOI:** 10.3389/fpsyg.2025.1693697

**Published:** 2025-11-19

**Authors:** Ebru Yıldırım, Mustafa Yusuf Kol, Mehmet Fatih Özkan, Ömer Faruk Doğru, Bahar Güntekin

**Affiliations:** 1 Neuroscience and Neurotechnology Center of Excellence (NÖROM), Gazi University, Ankara, Türkiye; 2 School of International Medicine, Istanbul Medipol University, Istanbul, Türkiye; 3 Department of Biophysics, School of Medicine, Istanbul Medipol University, Istanbul, Türkiye; 4 Clinical Electrophysiology, Neuroimaging and Neuromodulation Lab, Research Institute for Health Sciences and Technologies (SABITA), Neuroscience Research Center, Istanbul Medipol University, Istanbul, Türkiye

**Keywords:** EEG, video gaming, event-related oscillations (EROs), memory, low-frequency oscillations

## Abstract

**Introduction:**

Frequent exposure to video gaming induces changes in cognitive and perceptual functions and alterations in neural structure and functioning. While frequent video gaming has been associated with positive effects in cognitive-perceptual domains, it may concurrently exert adverse impact on social–emotional functioning. This study used event-related EEG brain oscillations to investigate the effect of frequent playing video games on the visual and auditory working memory processes.

**Methods:**

The study included 23 healthy young men participants, divided into frequent gamer and infrequent gamer groups based on their exposure to violent video games. An internet-based questionnaire was used for group classification, and the frequent gamer group consisted of participants who played more than 15 h of video games per week. EEG recordings were obtained during visual and auditory memory tasks, and participants’ anxiety levels were assessed using the State–Trait Anxiety Inventory. Event-related power spectrum and phase-locking analyses were conducted for delta, theta, and alpha frequencies.

**Results:**

No significant group differences were observed in behavioral performance and anxiety levels; however, there were notable electrophysiological differences. The frequent gamers exhibited lower and shorter visual delta responses compared to infrequent gamers. A left-hemisphere dominance for the frequent gamers was observed in auditory theta and alpha power, particularly in the parietal and occipital regions. Additionally, the frequent gamers showed reduced visual alpha power in posterior regions and less increase in auditory lower alpha phase-locking.

**Discussion:**

In conclusion, the observed alterations in low-frequency event-related oscillations suggest that the frequent gamers employ distinct neurocognitive strategies during memory tasks. These strategies may reflect enhanced efficiency in specific domains such as attention and memory, despite similar behavioral performance.

## 1 Introduction

While playing video games has become a popular leisure activity, leading individuals to spend significant time engaged in gameplay (Clement, 2024). This extensive exposure to video games has been shown to affect both neural structures and functions (Kühn and Gallinat, 2014). Literature frequently reports both positive and negative effects of video gaming. Several studies have highlighted its beneficial impact on cognitive and perceptual domains (Kühn and Gallinat, 2014; Boot et al., 2008; Boot et al., 2011; Green and Bavelier, 2012; Anguera et al., 2013; Wilms et al., 2013; Hilla et al., 2020). On the other hand, adverse outcomes have also been observed in the socio-emotional domain (Browne and Hamilton-Giachritsis, 2005). Evidence suggests that video gaming can enhance attention, memory, visuospatial, and motor skills; however, it has also been associated with increased aggression, addictive behaviors, and reductions in empathy and prosocial conduct (Bavelier et al., 2011).

Behavioral outcomes among video game players have been shown to vary depending on the genre of the games played. While prosocial games have been associated with increased helpfulness and reduced tendencies toward harmful behavior, the opposite pattern has been reported for action games (Saleem et al., 2012). The potential impact of violent video games on aggression remains one of the most widely studied and hotly debated topics in literature. It has been suggested that repeated exposure to violent content may lead to desensitization to violence, which in turn could increase aggressive tendencies and diminish prosocial behaviors (Brockmyer, 2015). However, other studies have produced more nuanced findings. Ferguson (2007) reported that while video game play may elevate aggressive thoughts, it does not necessarily translate into overt aggressive behavior. This meta-analysis study showed no strong link between action game play and increased aggression (Ferguson, 2007). Similarly, a study comparing spontaneous brain activity in individuals who played violent video games with that of control participants found no significant differences, suggesting that the potential harms of such games may be overstated. Despite prolonged exposure, no adverse effects were observed on core cognitive functions such as working memory (Pan et al., 2018). The literature focuses on the potential harms of video games, particularly action games, while empirical investigations into their possible benefits remain scarce. Although recent years have witnessed efforts to address this research gap, the literature still lacks comprehensive evidence regarding the positive aspects of video game engagement.

A growing body of literature has begun to highlight the potential benefits of video games, particularly in educational contexts, through the repurposing of existing games, the enhancement of their positive effects, and their potential to promote individual and societal well-being. One such genre, Massively Multiplayer Online Role-Playing Games (MMORPGs), has been shown to enhance well-being and foster social development when played cooperatively and within reasonable time limits (Longman et al., 2009). However, excessive gameplay, ranging from 44 to 82 h per week, has been associated with psychological symptoms such as anxiety, depression, and stress. These adverse effects, however, may be mitigated through adequate social support and moderation in gameplay duration (Longman et al., 2009). A more recent development in the gaming landscape is the emergence of “Exergame,” which integrates physical activity into gameplay. These games aim to reduce physiological symptoms by simulating traditional exercise within an entertainment framework, thereby merging health promotion with interactive digital media (Halbrook et al., 2019). Research on the cognitive benefits of video games has demonstrated that players often exhibit superior visual attention and spatial distribution skills compared to non-players. Green and Bavelier (2003) found that video game players could sustain attention even during demanding tasks that typically increase susceptibility to distractions.

Furthermore, the number of visual items accurately perceived without error was significantly higher among gamers (Green and Bavelier, 2003). Visuospatial abilities are particularly enhanced in individuals who engage with video games, especially action games. Remarkably, even after a brief period of 5–10 h of action gameplay, improvements in visuomotor coordination were observed, effects not observed in non-gaming control groups (Li et al., 2016). These findings suggest the potential utility of video games in training contexts. One such application is in laparoscopic surgery, where advanced visuospatial skills, attention, and depth perception are critical. Previous research has indicated that individuals with gaming experience outperform non-gamers in laparoscopic simulation tasks (Sammut et al., 2017).

Neuroimaging techniques have been employed to investigate neural and functional brain changes associated with video game play. Findings from these studies suggest that video gaming is particularly linked to alterations in visuospatial cognition and attentional processes (Kühn et al., 2019). However, executive functions, memory, and overall cognition results are more complex and less conclusive, making generalizations difficult. Several methodological limitations hinder a clear understanding of how video games influence cognition, brain structure, and function. These limitations include the lack of standardized protocols, insufficiently defined classifications of video game genres, and the failure to isolate cognitive ability as an independent variable within study designs (Kühn et al., 2019).

Among action video games, the most popular subgenre is First-Person Shooter (FPS) games. These games, played from the protagonist’s point of view, typically involve a high degree of fantastical or realistic violence (Spence and Feng, 2010). Since the player’s primary goal is to identify potential threats, effectively scanning the visual field is essential. With advancements in technology enabling navigation across expansive in-game maps, players must remain constantly vigilant to detect and respond to emerging threats. Success in these games depends on the player’s ability to visually distinguish allies from enemies, classify distant or ambiguous moving targets, track relevant cues, and make rapid, strategic decisions (Spence and Feng, 2010). Importantly, this occurs within high-stress environments that demand swift and precise reactions. The appropriateness of a player’s response to sudden, unexpected stimuli hinges on the integrated functioning of perception, cognitive processing, and motor execution (Spence and Feng, 2010). Moreover, it has been demonstrated that players of action video games exhibit enhanced visual selective attention in several dimensions (Green and Bavelier, 2003). Neurophysiological evidence suggests that, in the presence of dynamic distractors, players show reduced activation in motion-sensitive visual areas, indicating more efficient filtering of irrelevant information. Additionally, reduced fronto-parietal connectivity during attentional tasks in gamers implies that these individuals require less cognitive effort to discriminate between relevant and irrelevant stimuli, thus performing demanding attentional functions with greater ease (Bavelier et al., 2012).

It is well-established that action-oriented video games, such as first-person shooters (FPS), enhance attentional and perceptual abilities; however, the underlying neurophysiological mechanisms responsible for these improvements have not yet been thoroughly investigated. Studies employing electroencephalography (EEG) remain limited in number and predominantly focus on event-related potentials (ERPs). In a previous study, participants who regularly played action video games and those who did not were instructed to detect targets appearing either at central or peripheral locations. This task elicited steady-state visual evoked potentials (SSVEPs) and ERPs. Findings indicated that gamers performed the task both more rapidly and more accurately. Moreover, non-target peripheral stimuli elicited more strongly suppressed SSVEPs in gamers, and target-related P300 amplitudes were reported to be significantly higher (Mishra et al., 2011).

Event-related brain oscillations in EEG are derived through the analysis of event-related potentials (ERPs) in both the time and frequency domains. These oscillations are important for understanding brain functions (Başar et al., 2001). Despite the growing interest in the cognitive effects of video games, research utilizing EEG to investigate their potential benefits remains limited. Moreover, existing studies have predominantly focused on event-related potentials, rather than event-related oscillations. Investigating the effects of video games on brain mechanisms through event-related brain oscillations may yield more comprehensive insights. The present study aimed to examine the impact of playing violent action video games on visual and auditory memory processes by analyzing event-related EEG brain oscillations in frequent gamers and infrequent gamers. As outlined above, we hypothesize that such games may contribute to enhancing visual and auditory memory-related skills. By exploring the influence of violent action games from visual and auditory memory perspectives, this study seeks to provide a more comprehensive contribution to the literature and inform future research directions. Furthermore, it aspires to support existing findings suggesting that video games can be leveraged for educational purposes. Given the widespread use of computer games in society, understanding their effects on cognitive functions is of great relevance, not only from a scientific standpoint but also from the perspective of users.

## 2 Materials and methods

### 2.1 Participants

In total, 23 participants were enrolled in the study, all of whom were undergraduate students. They formed the following two groups: frequent gamers and infrequent gamers. An internet-based questionnaire was administered to determine the participants for the frequent gamer and infrequent gamer groups, as considered by previous studies (Engelhardt et al., 2011). In the questionnaire, participants were asked to list the five video games they played most frequently and indicate the number of hours they played these games per week for each of the five games. Afterward, the scores for five games were summed (Engelhardt et al., 2011; Anderson and Dill, 2000). Participants whose total scores exceeded a predetermined threshold were assigned to the gamer group, with our study defining this threshold as 15 h of gameplay per week (Wilms et al., 2013; Hazarika et al., 2018). The participants whose total weekly gameplay exceeded 15 h were assigned to the frequent gamer group. Those who played less than 15 h per week were assigned to the infrequent gamer group. The frequent gamer group consisted of 12 healthy young men (age mean: 19.75 ± 1.60) aged 18–30, who had no psychiatric and/or neurological disorders, were not using any psychiatric and/or neurological medications, and regularly played violent video games. The infrequent gamer group included 11 healthy young men (age mean: 20.09 ± 1.30) aged 18–30, who did not have any psychiatric and/or neurological diseases, were not using any psychiatric and/or neurological drugs, and did not regularly play violent video games. There were no participants who had no gaming experience in the infrequent gamer group. All participants had at least some prior gaming experience. No statistically significant difference existed between groups for the age factor (*p* = 0.489). Subjects with epilepsy, a history of head trauma, use of medications affecting cognitive performance, or alcohol and drug abuse were excluded from the study. The present study was approved by the local Ethical Committee (Ethical report no: 10840098–772.02-954). All participants signed the informed consent form.

### 2.2 Data collection tools

#### 2.2.1 Behavioral tests

In our study, an internet-based questionnaire was administered, informed by previous studies (Engelhardt et al., 2011). In the questionnaire, participants were asked to list the five video games they played most frequently, indicate the number of hours they played these games per week, and rate of the violence in terms of the contents and graphics of these games on a scale ranging from 1 to 7 (1 = Non-violent, 7 = Highly violent) (Engelhardt et al., 2011). Afterward, the scores for five games were summed and noted as total hours spent playing games weekly for each participant. Furthermore, the scores for violence in terms of content and graphics were averaged for five games and these values were noted as the mean violence of content and graphics.

State–Trait Anxiety Inventory scale (STAI-TX) was administered to participants before EEG recording. This scale, developed by Spielberger (1970), is a Likert-type measure used to assess individuals’ anxiety levels. The inventory consists of two sections, each containing 20 items. The first section measures state (situational) anxiety intensity (STAI-TX1), while the second section assesses trait (general) anxiety intensity (STAI-TX2). Each question is rated on a 4-point scale ranging from 1 (“Not at all”) to 4 (“Completely”), and participants are asked to select the response that best reflects their feelings. The theoretical scores obtained from both scales range from 20 to 80. Higher scores indicate a higher level of anxiety, while lower scores reflect a lower level of anxiety (Spielberger, 1970).

#### 2.2.2 EEG

##### 2.2.2.1 EEG data recording

The EEG recordings were conducted in an isolated and dimly lit room. The EEG data were recorded using a BrainAmp 32-Channel MR Plus system (Brain Product, Germany) with a bandpass filter range of 0.01–250 Hz and a sampling rate of 500 Hz. The elastic cap (Easy Cap) with multirodes (Brain Product, Germany) was used for EEG recordings. The electrode placement was according to the international 10–20 system. Two linked electrodes (A1 + A2) were placed on the anterior of the earlobes as reference electrodes. Furthermore, electrooculography (EOG) recordings were performed from two electrodes (vertically and horizontally). The impedance values of all electrodes were kept below 10 kΩ.

##### 2.2.2.2 Experimental design

**Figure 1:**
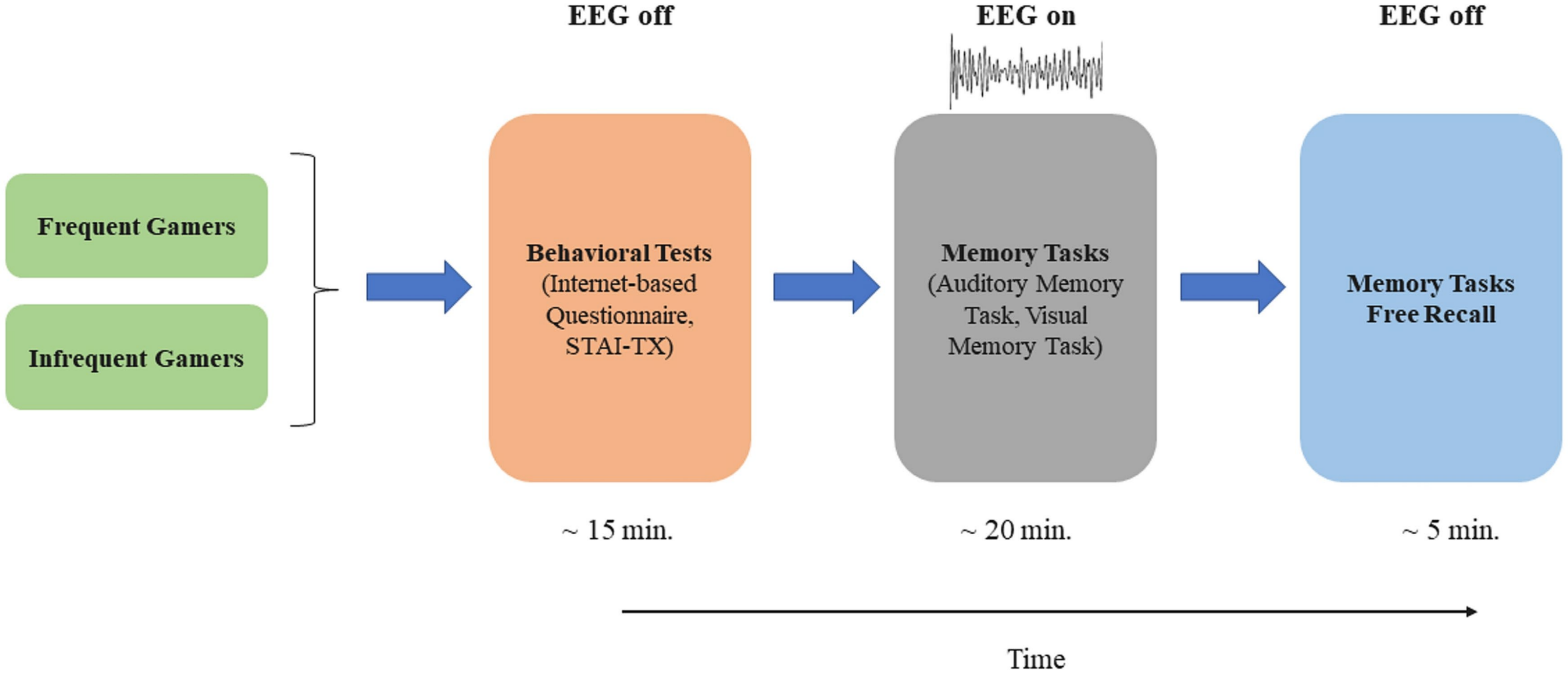
The schematic illustration of the experimental timeline.

In our study, auditory and visual memory tasks were administered to participants, and event-related EEG data were recorded simultaneously during these tasks. The memory tasks assessed working memory, involving both encoding and repeated maintenance of information. Figure 1 provides a schematic illustration of the experimental timeline. The auditory and visual tasks were designed using E-Prime software. In the auditory memory task, words selected from the Öktem Verbal Memory Test word list were used as stimuli (Tanör, 2011). The selected words comprised familiar nouns with comparable syllable lengths and were commonly used in Turkish, thus ensuring consistency across the stimuli. The auditory stimuli (i.e., the sounds of these words) were recorded in an isolated room by a native Turkish speaker and subsequently standardized in terms of decibel level and duration using Audacity software (Audacity^®^), thus ensuring consistency across stimuli. Images selected from the Boston Naming Test were used as stimuli for the visual memory task (Kaplan et al., 2001) and consisted of black-and-white line drawings. The images were selected based on simplicity, clarity, and high familiarity, to ensure that participants could readily recognize them.

In the auditory memory paradigm, participants were presented with auditory stimuli and were instructed to concentrate on and memorize the presented words, regardless of their order. The participants viewed a black screen with no fixation mark or other visual elements while memorizing the auditory stimuli. In the visual memory paradigm, the visual stimuli were presented to participants on a full-screen monitor measuring 47.5 × 26.8 cm, with a refresh rate of 60 Hz, positioned 90 cm from the participants. Participants were instructed to concentrate on and memorize presented images, regardless of the order of presentation. Immediately following the completion of each paradigm, participants were asked to report the items they remembered, during which no EEG recording took place. The number of successfully recalled (free recall) items for each memory task is used as a memory performance score. Each of the auditory and visual memory paradigms consisted of 25 stimuli, each presented three times in a total of 75 stimuli. The stimuli were presented randomly for 1 s each, with inter-stimulus intervals ranging from 3 to 5 s. Before beginning the tasks, a practice session was conducted to ensure participants fully understood the instructions.

### 2.3 EEG analysis

Event-related EEG data recorded during the auditory and visual memory tasks were analyzed using BrainVision Analyzer software (Brain Products, Munich, Germany). Before analyzing event-related oscillations, raw EEG data underwent preprocessing to remove artifacts. First, the continuous EEG signal was band-pass filtered between 0.01 and 60 Hz using Infinite Impulse Response (IIR) filters. Subsequently, Independent Component Analysis (ICA) was applied to identify and remove components related to eye movements and blinks. The EEG data were then segmented into epochs relative to stimulus onset. Epoch lengths were set differently for delta and theta/alpha bands, as delta is a slower frequency compared to theta and alpha and therefore requires longer time windows to accurately capture its temporal dynamics in wavelet-based analyses (Herrmann et al., 2005). For the analysis of theta and alpha frequency bands, the epoch ranges were defined from 1,000 ms before to 1,000 ms after stimulus onset. For the delta frequency band, this range was extended from 3,000 ms before to 3,000 ms after stimulus onset. Subsequently, manual artifact rejection was performed, and epochs containing artifacts, such as muscle activity and electrode noise, were excluded. Finally, event-related power spectrum and phase-locking analyses were conducted on the preprocessed artifact-free EEG data.

#### 2.3.1 Event-related power spectrum analysis

Power spectrum analysis-one of the most common time-frequency techniques used in event-related oscillation (ERO) research-examines changes in EEG spectral power following stimulus presentation. At the present study, the event-related power spectrum was analyzed in the delta (1–4 Hz), theta (4–7 Hz), and alpha (8–13 Hz) frequency bands. Power spectrum analyses were conducted using a continuous wavelet transform with Gabor normalization, a wavelet width of three cycles, and a baseline correction (−500 to −300 ms). This baseline period was selected to provide sufficient temporal proximity to the event while minimizing contamination from anticipatory or early post-stimulus activity, consistent with previous studies (Tülay et al., 2020; Güntekin et al., 2019; Güntekin et al., 2023; Çelik et al., 2024; Çelik et al., 2025; Dilek et al., 2023). In the power spectrum analysis, the Morlet wavelet (Morlet complex) transform was applied to each preprocessed artifact-free epoch. Then, power spectra of all those epochs were averaged. Subsequently, the event-related power spectrum values were computed based on grand averages for each frequency band. Event-related delta power values were calculated for two distinct time windows: 0–700 ms and 1,000–1,600 ms. Theta power values were calculated for the interval of +50 to +300 ms. Alpha power spectrum values were calculated for two-time intervals (0–200 ms and 200–400 ms) and two sub frequency bands (8–10 Hz and 10–13 Hz). These resulting power values were used for the statistical analysis.

#### 2.3.2 Event-related phase-locking analysis

Phase-locking-a crucial measure for evaluating the neurophysiological oscillatory mechanisms underlying cognitive processes-computes the coherence between the phase angles of the stimuli (Güntekin et al., 2022). The phase-locking value ranges from 0 to 1, where 1 indicates maximal phase consistency (i.e., all stimuli evoke identical phase responses), and 0 reflects complete phase randomness (i.e., a purely non-phase-locked response). At the present study, event-related phase-locking was analyzed in the delta (1–4 Hz), theta (4–7 Hz), and alpha (8–13 Hz) frequency bands. Phase-locking analyses were conducted using a continuous wavelet transform with Gabor normalization and a wavelet width of three cycles. In the phase-locking analysis, the Morlet wavelet (Morlet complex) transform was applied to each preprocessed artifact-free epoch, and then, the results of all epochs were averaged. Subsequently, the phase-locking values were computed based on grand averages for each frequency band. Event-related delta phase-locking was analyzed within two-time intervals: 0–700 ms and 1,000–1,600 ms post-stimulus. Theta phase-locking was calculated for the 50–250 ms window. Alpha phase-locking was analyzed across two time intervals (0–200 ms and 200–400 ms) and two sub frequency bands: 8–10 Hz and 10–13 Hz. The resulting phase-locking values were used for statistical analysis.

### 2.4 Statistical analysis

Statistical analyzes were conducted using repeated measures ANOVA in Jamovi (2.6.2 version) software. Each of the ERO parameters—delta power spectrum, delta phase-locking, theta power spectrum, theta phase-locking, alpha power spectrum, and alpha phase-locking—was analyzed separately. ANOVA tests were applied independently for the auditory and visual memory tasks, resulting in a total of 20 separate ANOVA analyses. These included ANOVA tests for the ERO delta power spectrum in the first- and second-time intervals, ERO delta phase-locking in the first- and second-time intervals, ERO theta power spectrum, ERO theta phase-locking, ERO alpha power spectrum in the lower and upper sub frequency bands, and ERO alpha phase-locking in the lower and upper sub frequency bands, each for both auditory and visual memory tasks. The ANOVA design for delta and theta ERO responses included: 2 groups (frequent gamer, infrequent gamer) × 5 electrode locations [frontal (F3–F4), central (C3–C4), parietal-1 (P3–P4), parietal-2 (P7–P8), and occipital (O1–O2)] × 2 hemispheres (left, right). For alpha ERO responses, the design included: 2 groups × 2 time intervals [first (0–200 ms), second (200–400 ms)] × 5 electrode locations × 2 hemispheres. Greenhouse–Geisser adjusted *p*-values were reported. Tukey test was employed for post-hoc multiple comparison corrections. Additionally, exploratory analyses were conducted to compare behavioral outcomes—task performance, scores from the internet-based questionnaire, and anxiety levels—between the groups using the Mann–Whitney U test. All data were assessed for normality using the Shapiro–Wilk test. Parametric or non-parametric analyses were applied depending on whether the data met the normality assumption. The statistical significance threshold was set at *p* < 0.05.

## 3 Results

### 3.1 Behavioral results

The behavioral results, including task performance, scores from the internet-based questionnaire, and STAI-TX scores, are presented in Table 1. As previously mentioned, the Mann–Whitney U test was used to compare behavioral scores between the frequent gamer and infrequent gamer groups.

**Table 1:** Behavioral results of frequent gamer and infrequent gamer groups.

Variable	Frequent gamer (*N* = 12) Mean ± SD	Infrequent gamer (*N* = 11) Mean ± SD	*P*
Total hours spent playing games weekly	38.60 ± 20.60	4.00 ± 2.96	**<0.001** ^**a**^
The violence of the contents	3.51 ± 1.70	3.85 ± 2.28	0.644^a^
The violence of graphics	3.10 ± 1.91	3.79 ± 2.22	0.372^a^
STAI-TX1	26.80 ± 6.24	31.50 ± 10.20	0.265^a^
STAI-TX2	36.20 ± 8.47	36.00 ± 6.83	0.805^a^
Remembered auditory items	20.00 ± 4.57	19.40 ± 4.90	0.577^a^
Remembered visual items	17.70 ± 3.47	17.00 ± 4.05	0.709^a^

^a^Mann–Whitney *U* test; SD, Standard deviation; STAI-TX1: State-Trait Anxiety Inventory scale situational anxiety score; STAI-TX2: State-Trait Anxiety Inventory scale general anxiety score. Bold values indicate statistically significant results (*p* < 0.05).

Compared to the infrequent gamer group, the frequent gamer group had significantly higher total weekly hours spent playing games (*p* < 0.001). However, no significant differences were found between the groups in mean scores for the violence of content (*p >* 0.05) and graphics (*p >* 0.05) in the games they played. Similarly, anxiety levels did not differ significantly between the groups (*p >* 0.05 for STAI-TX1; *p >* 0.05 for STAI-TX2). Furthermore, there was no significant difference between the frequent gamer and infrequent gamer groups in the number of remembered items for both auditory (*p >* 0.05) and visual tasks (*p >* 0.05).

### 3.2 Event-related power spectrum results

#### 3.2.1 Delta power

The group main effect was not statistically significant for delta power within the early 0–700 ms time interval during the auditory memory task. Similarly, the group main effect was not statistically significant for delta power during the visual memory task.

**Figure 2:**
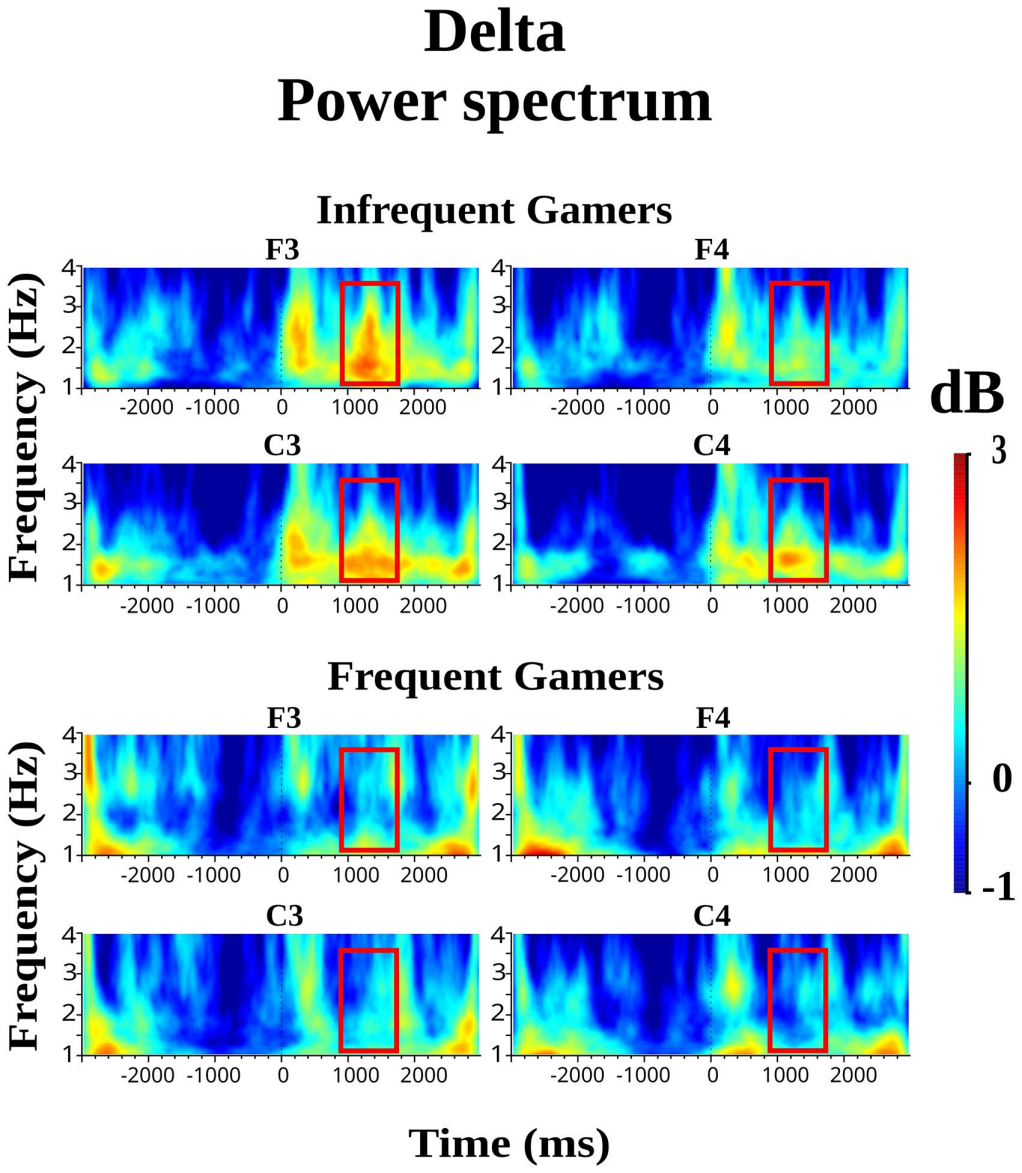
Grand average of delta power during the visual memory tasks at the F3, F4, C3, and C4 electrodes for the frequent gamer and infrequent gamer groups.

However, the group main effect was not statistically significant for delta power within the late 1,000–1,600 ms time interval during the auditory memory task. In contrast, the group main effect was close to the significance level for delta power during the visual memory task [*F*_(df = 1,21)_ = 3.81; *p* = 0.064; *η*_p_^2^ = 0.154]. During the late 1,000–1,600 ms time interval of the visual memory task, the infrequent gamer group exhibited higher delta power than the frequent gamer group. Figure 2 presents the grand average of delta power during visual memory tasks at the infrequent and frequent gamer groups’ F3, F4, C3, and C4 electrodes (for illustration across all scalp electrodes, see Supplementary Image S1). As shown in Figure 2, delta power differed significantly between the groups in the late (1000–1,600 ms) time interval of the visual memory task, the infrequent gamer group exhibited greater delta power than the frequent gamer group.

#### 3.2.2 Theta power

The infrequent gamer group exhibited higher theta power than the frequent gamer group during both auditory and visual memory task; however, this group difference was not statistically significant. The Location × Hemisphere × Group interaction was statistically significant only for the auditory memory task [*F*_(df = 2.68,56.37)_ = 3.413; *p* = 0.028; *η*_p_^2^ = 0.140]. *Post hoc* analyses revealed that at the parietal-2 locations, the frequent gamer group exhibited higher theta power in the left hemisphere than in the right hemisphere [*t*(21.0) = 2.68, *p* = 0.014].

#### 3.2.3 Alpha power

**Figure 3:**
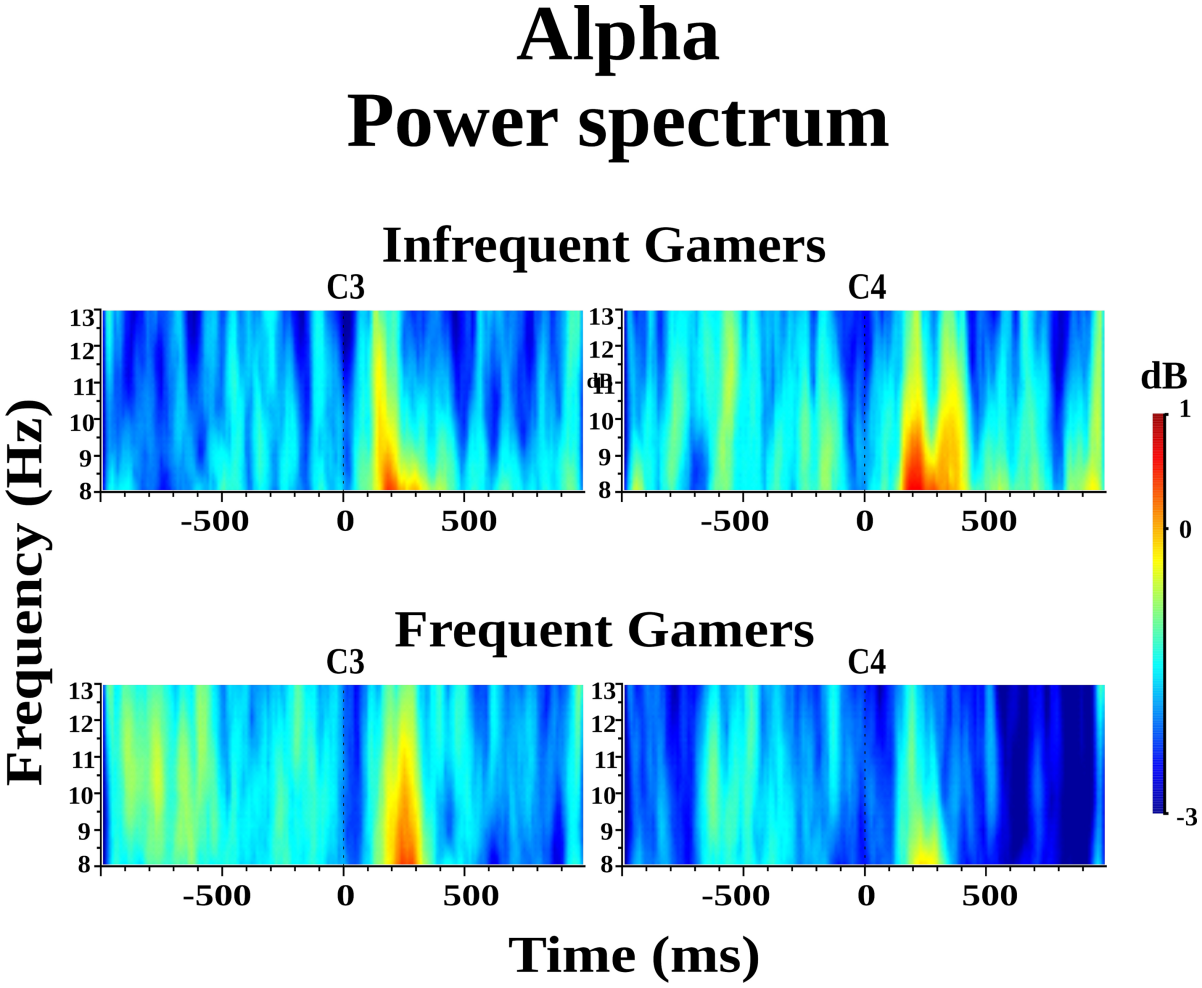
Grand average of lower alpha power within the 8–10 Hz sub frequency band during the auditory memory task at the C3 and C4 electrodes for the frequent gamer and infrequent gamer groups.

The group difference in lower alpha power within the 8–10 Hz sub frequency band during the auditory memory task was not statistically significant. However, the Hemisphere × Group interaction was statistically significant for the auditory memory task [*F*_(df = 1,21)_ = 10.214; *p* = 0.004; *η*_p_^2^ = 0.327] (Figure 3). *Post hoc* comparisons revealed that, within the frequent gamer group, lower alpha power in the left hemisphere was higher than in the right hemisphere [*t*(21.0) = 2.98, *p* = 0.033] (for illustration across all scalp electrodes, see Supplementary Image S2).

**Figure 4:**
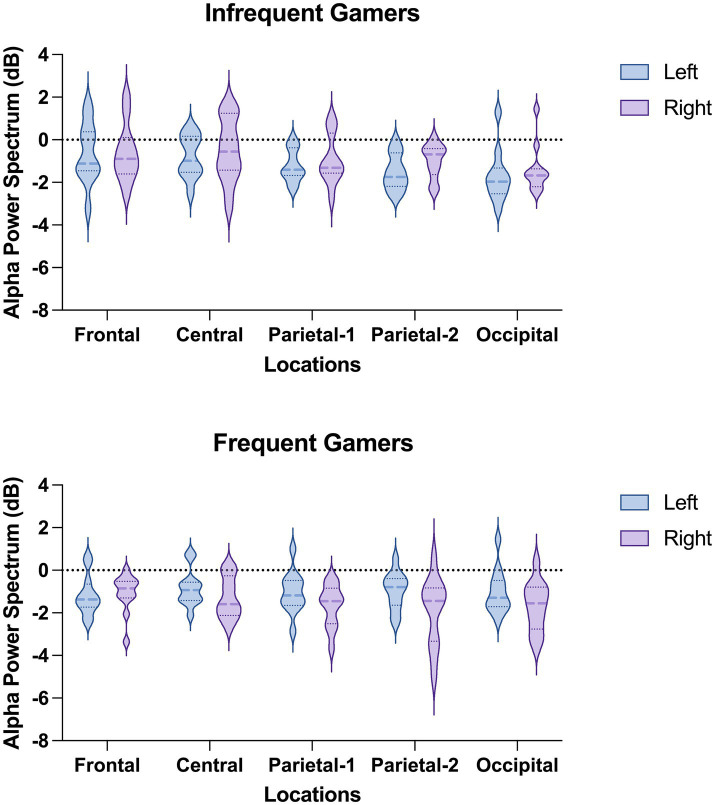
The violin graphs of the Location × Hemisphere × Group interaction in lower alpha power within the 8–10 Hz sub frequency band during the auditory memory task.

The Location × Hemisphere × Group interaction was statistically significant for the auditory memory task [*F*_(df = 3.25,68.30)_ = 3.541; *p* = 0.016; *η*_p_^2^ = 0.144] (Figure 4). Post-hoc analyses revealed that, in the frequent gamer group, lower alpha power in the left hemisphere was higher than in the right hemisphere at the parietal-1 [*t*(21.0) = 2.98, *p* = 0.014], parietal-2 [*t*(21.0) = 3.08, *p* = 0.006], and occipital locations [*t*(21.0) = 3.70, *p* = 0.001]. The Time interval × Hemisphere × Group interaction was close to the significance level for the auditory memory task [*F*_(df = 1,21)_ = 3.886; *p* = 0.062; *η*_p_^2^ = 0.156]. In the frequent gamer group, lower alpha power in the left hemisphere was higher than in the right hemisphere during both the first (0–200 ms) and second (200–400 ms) time intervals.

**Figure 5:**
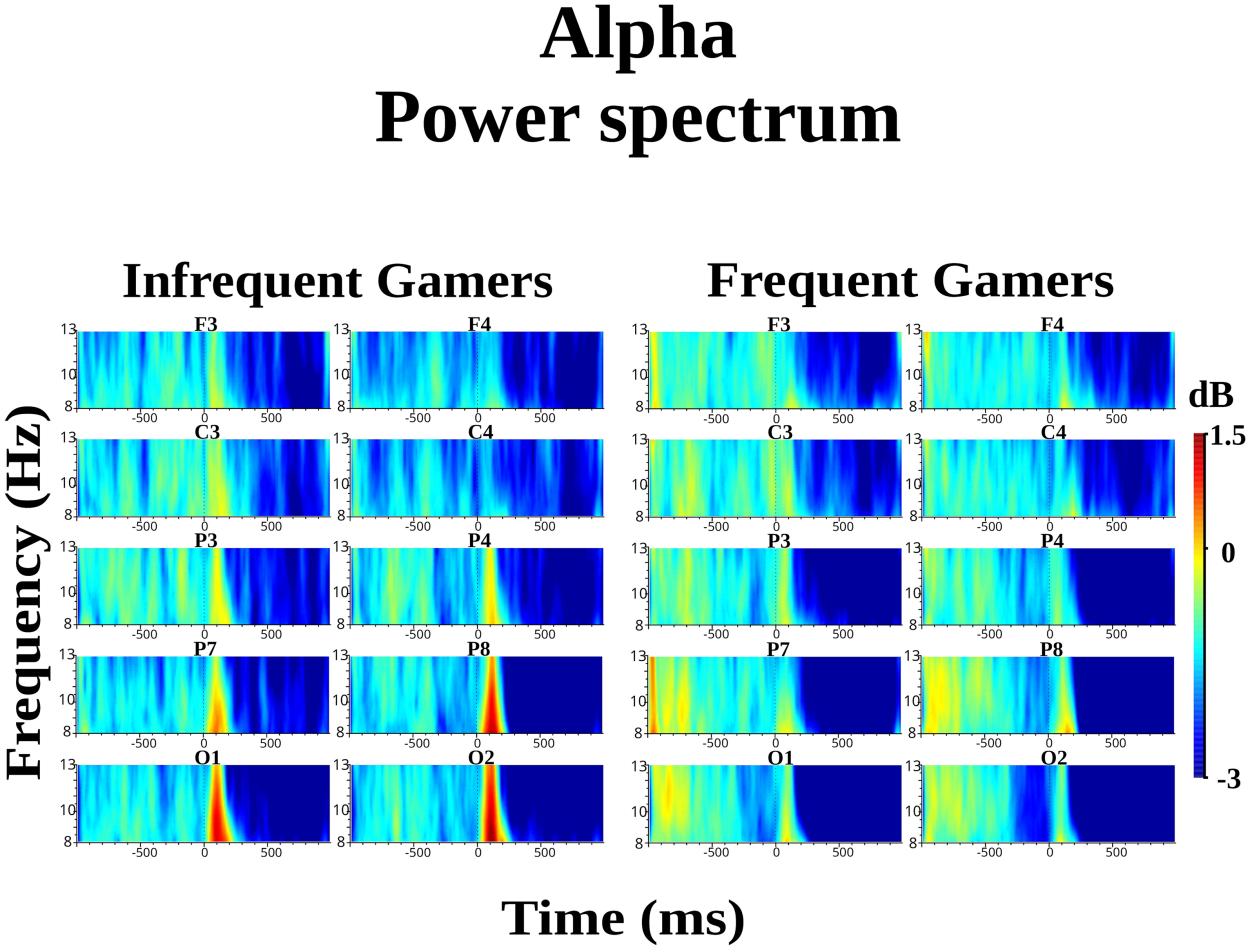
Grand average of lower alpha power within the 8–10 Hz sub frequency band during the visual memory task for the frequent gamer and infrequent gamer groups.

The group difference was not statistically significant for lower alpha power in the 8–10 Hz sub frequency band during the visual memory task. However, the groups differed significantly depending on location [*F*_(df = 2.11,44.39)_ = 3.313; *p* = 0.043; *η*_p_^2^ = 0.136]. At the parietal-2 and occipital locations, the infrequent gamer group exhibited higher lower alpha power than the frequent gamer group (Figure 5).

The group difference in upper alpha power within the 10–13 Hz sub frequency band during the auditory memory task was not statistically significant. However, the Hemisphere × Group interaction was statistically significant for the auditory memory task [*F*_(df = 1,21)_ = 7.528; *p* = 0.012; *η*_p_^2^ = 0.264]. *Post hoc* comparisons revealed that, within the frequent gamer group, upper alpha power in the left hemisphere was higher than in the right hemisphere [*t*(21.0) = 2.74, *p* = 0.012]. Additionally, the frequent gamer group exhibited higher upper alpha power than the infrequent gamer group in the left hemisphere [*t*(21.0) = 2.70, *p* = 0.013].

The group difference in upper alpha power within the 10–13 Hz sub frequency band during the visual memory task was not statistically significant. However, the effect was close to the significance level depending on location [*F*_(df = 2.22,46.57)_ = 2.919; *p* = 0.059; *η*_p_^2^ = 0.122]. The infrequent gamer group exhibited higher upper alpha power at the occipital locations than the frequent gamer group (Figure 5).

### 3.3 Event-related phase-locking results

#### 3.3.1 Delta phase-locking

The group main effect was not statistically significant for delta phase-locking within the early 0–700 ms time interval during the auditory memory task. Similarly, the group main effect was not statistically significant for delta phase-locking during the visual memory task.

**Figure 6:**
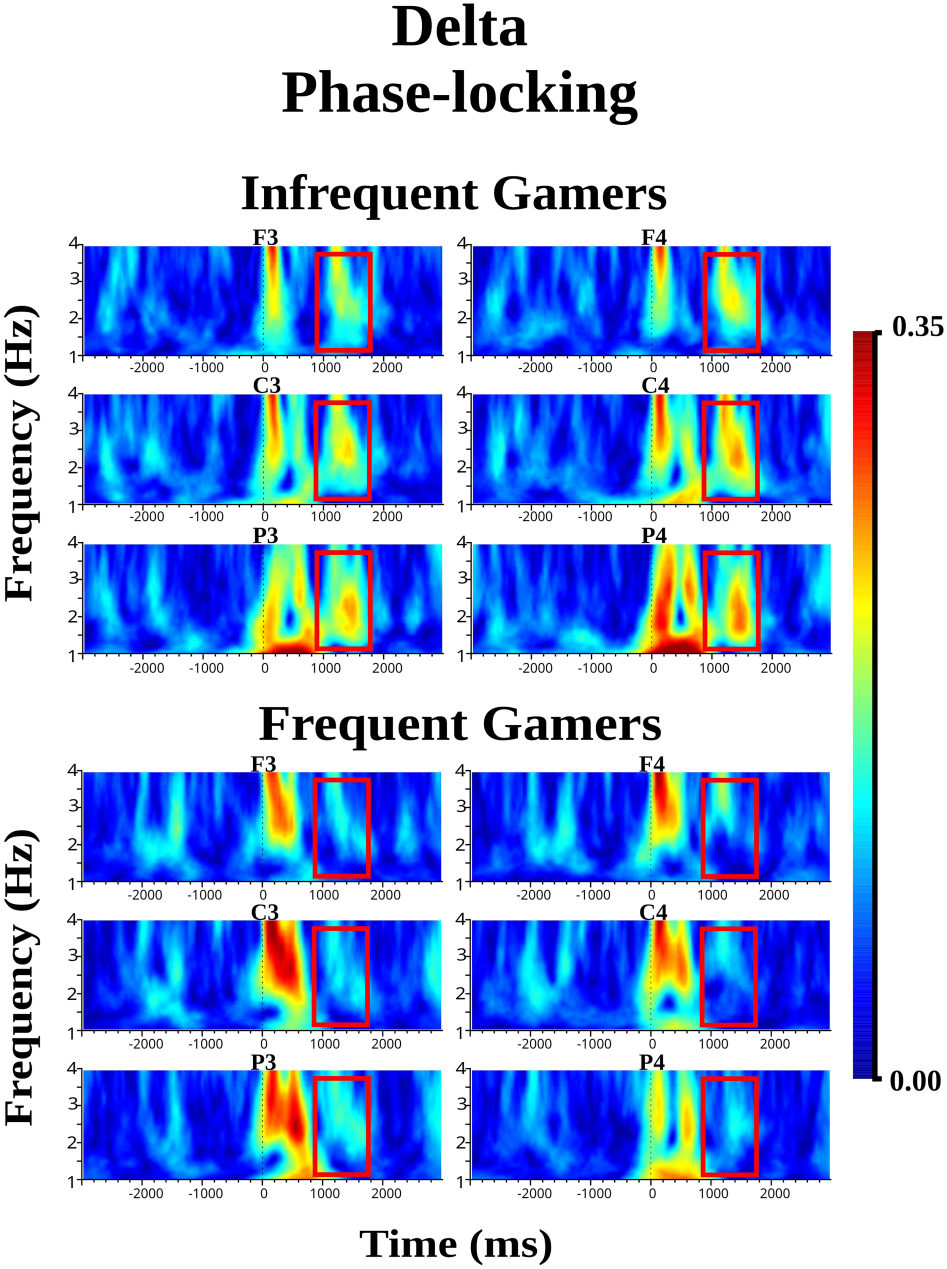
Grand average of delta phase-locking during the visual memory task for the frequent gamer and infrequent gamer groups.

The group main effect was not statistically significant for delta phase-locking within the late 1,000–1,600 ms time interval during the auditory memory task. However, the group main effect was close to the significance level for delta phase-locking during the visual memory task [*F*_(df = 1,21)_ = 3.98; *p* = 0.059; *η*_p_^2^ = 0.159]. During the late 1,000–1,600 ms time interval of the visual memory task, the infrequent gamer group exhibited higher delta phase-locking than the frequent gamer group. Figure 6 presents the grand average of delta phase-locking during the visual memory tasks at the frontal, central, and parietal electrodes for the infrequent gamer and frequent gamer groups. As shown in Figure 6, delta phase-differed significantly between the groups in the late (1000–1,600 ms) time interval of the visual memory task, the infrequent gamer group exhibited greater delta phase-locking than the frequent gamer group (for illustration across all scalp electrodes, see Supplementary Image S3).

#### 3.3.2 Theta phase-locking

The infrequent gamer group exhibited higher theta phase-locking than the frequent gamer group during the auditory memory task; however, this group difference was not statistically significant. Similarly, the group difference was not statistically significant for the visual memory task.

#### 3.3.3 Alpha phase-locking

**Figure 7:**
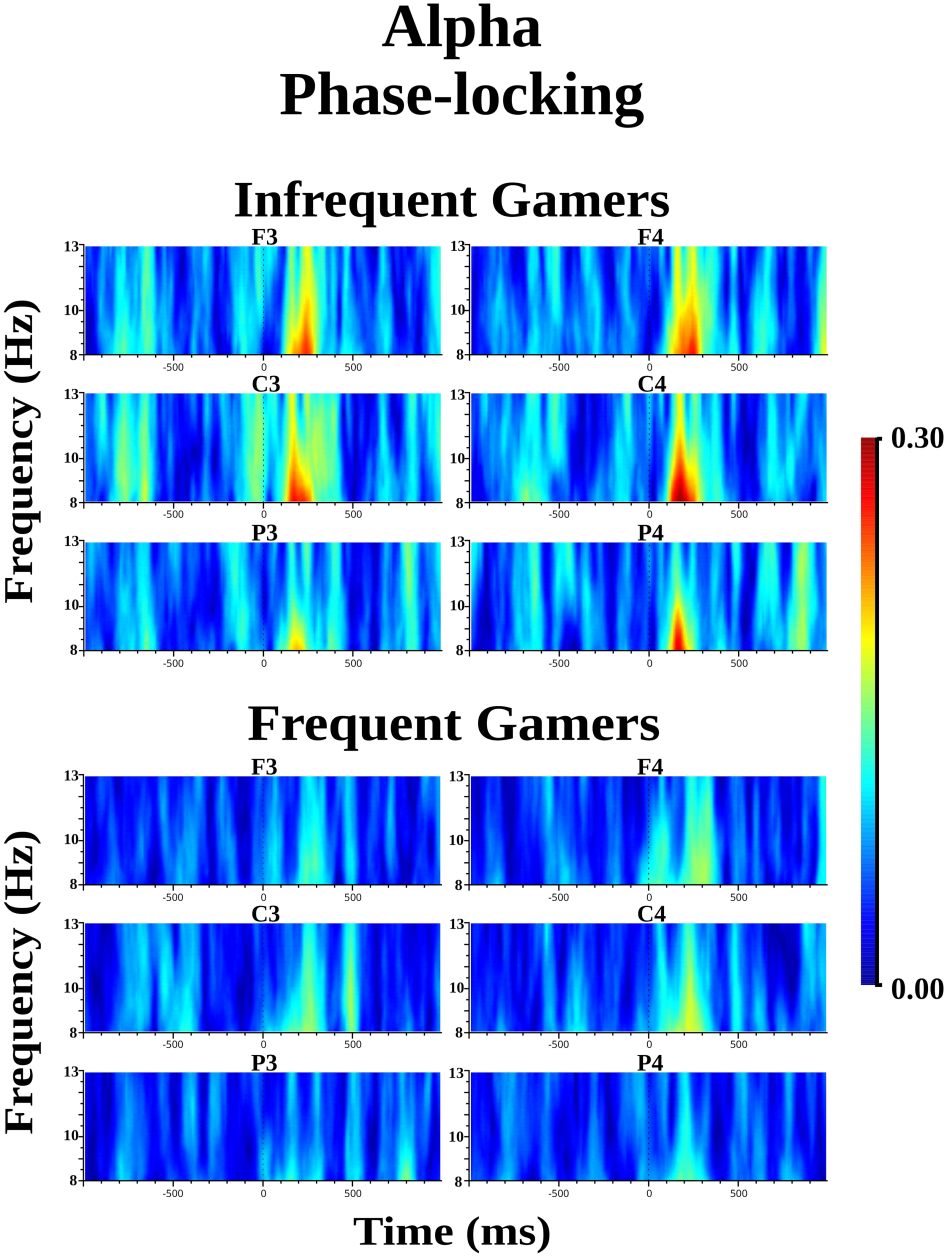
Grand average of alpha phase-locking during the auditory memory task for the frequent gamer and infrequent gamer groups.

The infrequent gamer group exhibited increased alpha phase-locking compared to the frequent gamer group in the 8–10 Hz sub frequency band during the auditory memory task. However, the difference between groups was not statistically significant for the lower phase-locking in the 8–10 Hz sub frequency band. The Time interval × Group interaction was close to the significance level for the auditory memory task [*F*_(df = 1,21)_ = 3.595; *p* = 0.072; *η*_p_^2^ = 0.146] (Figure 7). In the second time interval (200–400 ms), the infrequent gamer group exhibited higher alpha phase-locking than the frequent gamer group in the 8–10 Hz sub frequency band [*t*(21.0) = 2.28, *p* = 0.033] (for illustration across all scalp electrodes, see Supplementary Image S4). Conversely, the group difference in lower alpha phase-locking within the 8–10 Hz sub frequency band was not statistically significant during the visual memory task. No statistically significant group differences were observed in upper alpha phase-locking (10–13 Hz sub frequency band) during the auditory and visual memory tasks.

## 4 Discussion

Our study investigated event-related oscillatory (ERO) responses in the frequent gamers, who frequently play video games, and in infrequent gamers, who play infrequently, during visual and auditory memory tasks. This is the first study in the literature to compare event-related delta, theta, and alpha responses between these groups in visual and auditory memory tasks. Despite similar task performance, anxiety levels, and behavioral results between frequent gamers and infrequent gamers, our findings revealed significant electrophysiological differences. Specifically, we found that; (I) the frequent gamers exhibited lower and shortened visual delta responses (both power and phase-locking) compared to infrequent gamers. (II) For auditory theta and alpha power, the frequent gamers showed left hemisphere dominance, particularly in the parietal and occipital regions. (III) The frequent gamers demonstrated lower visual alpha power in posterior regions. (IV) The frequent gamers exhibited less increased auditory lower alpha (8–10 Hz) phase-locking than infrequent gamers.

Previous ERP studies have demonstrated that individuals who regularly play video games exhibit weaker and shorter P300 responses during cognitive tasks compared to non-gamers (Duven et al., 2015; Shin et al., 2017; Stockdale et al., 2017; Xu et al., 2024). As is well-established, P300 responses elicited during cognitive paradigms (e.g., the oddball paradigm) involving functions such as attention and working memory are closely associated with low-frequency delta oscillation (Schürmann et al., 1995; Güntekin and Başar, 2016). Consistent with this, a review by Harmony (2013) reported evidence showing that with rising cognitive load, task-related EEG delta power tends to increase in magnitude. Video game play has been reported to have beneficial effects on visual processing and cognitive flexibility (Boot et al., 2008; Green and Bavelier, 2003; Castel et al., 2005; Uddin, 2021). Moreover, previous literature suggests that the frontoparietal network, associated with working memory, appears less active in gamers than non-gamers. This indicates that gamers may process information more efficiently, utilizing this network with less cognitive effort (Bavelier et al., 2012). Consistent with the existing literature, our study found that despite similar task performance in the visual memory paradigm, the infrequent gamer group exhibited prolonged and elevated delta responses extending into later time windows. In contrast, frequent gamers showed significantly shorter latency and reduced amplitude in visual delta responses. These findings suggest that frequent gamers may perform the same task with reduced cognitive effort and greater efficiency. Furthermore, the fact that group differences emerged exclusively in visual delta responses may indicate that frequent gamers process visual information more rapidly than infrequent gamers.

Hemispheric specialization plays a significant role in memory processes (Sauseng et al., 2004; Miller et al., 2018; Penner et al., 2022). The left hemisphere is predominantly associated with language, verbal information processing, and verbal memory functions (Sauseng et al., 2004; Ravizza et al., 2005; Salisbury and Taylor, 2012), whereas the right hemisphere is primarily involved in visual information processing and visuospatial memory processes (Sauseng et al., 2004; Penner et al., 2022; d'Esposito et al., 1998). Furthermore, previous research has identified the parietal regions as cortical structures supporting working memory, with the left parietal lobe implicated in phonological loop functions and the right parietal lobe supporting visuospatial sketchpad operations (Ravizza et al., 2005; d'Esposito et al., 1998; Sauseng et al., 2005). Event-related theta and alpha oscillations are critically involved in memory processes, with theta oscillations playing a particularly important role (Sauseng et al., 2004; Klimesch, 1999; Sauseng et al., 2010; Akiyama et al., 2017). It has been reported that theta and alpha oscillations synchronize during working memory tasks, accompanied by increased amplitudes in these frequency bands (Sauseng et al., 2004; Akiyama et al., 2017). Puma et al. (2018) reported that increases in theta and alpha power vary according to task completion performance. These differences may arise from variations in the availability of cognitive resources, differences in the strategies employed to accomplish the task, or interactions between these factors (Gulbinaite et al., 2014). In our study, despite similar task performance between groups, frequent gamers exhibited increased theta and alpha responses predominantly in the left hemisphere, particularly over parietal sites, during the auditory memory task, whereas no hemispheric differences were observed in infrequent gamers. These findings parallel previous work by Vourvopoulos et al. (2017), who reported hemispheric asymmetry and modulation of alpha/theta rhythms in hardcore gamers during motor imagery-based BCI tasks, suggesting that frequent video game play may induce domain-general adaptations in oscillatory brain dynamics, often without overt behavioral differences. This left-lateralized enhancement of theta and alpha power in auditory-verbal memory processes among frequent gamers may reflect the influence of frequent gaming, which could engender the recruitment of distinct cognitive resources and strategies, thereby inducing hemispheric specialization. Although it has been commonly reported that video game play enhances visuospatial processing and attention (Green and Bavelier, 2003; Castel et al., 2005; Dye and Bavelier, 2010) (because video games are mainly visual), our results revealed an increase in auditory ERO responses in frequent gamers. This suggests that the effects of video game play may not be limited to the visual modality, and may extend to auditory cognitive processing as well as visual cognitive processing. Zhang et al. (2017) reported a supramodal transfer effect, whereby experience in visually gaming strengthens domain-general cognitive control mechanisms, such as selective attention, cognitive flexibility, and working memory (Zhang et al., 2017). These cognitive resources are shared across sensory modalities and can be flexibly recruited during auditory perception and learning. This idea is supported by neuroimaging studies, which showed that working memory recruits both domain-general frontoparietal networks and domain-specific sensory areas (Li et al., 2014). Moreover, behavioral studies indicated that casual and action video games enhance executive functions, reasoning, and attentional control beyond strictly visual skills (Baniqued et al., 2013). This suggests that the increase of auditory ERO responses observed in our study likely arises from improvements in supramodal attentional and working memory networks developed through video gaming play, rather than from direct sensory-level adaptations within the auditory system.

As mentioned above, event-related alpha oscillations are closely associated with memory processes. Moreover, during working memory tasks, alpha responses-particularly in parieto-occipital regions-are known to increase with memory load (Jensen et al., 2002; Jensen et al., 2002). The increase of event-related alpha oscillations is generally linked to active maintenance of memory function, attentional control, and suppression of irrelevant information (Cooper et al., 2003; Jensen and Mazaheri, 2010; Bonnefond and Jensen, 2012; Wianda and Ross, 2019). In the present study, the frequent gamer group exhibited a smaller increase in event-related alpha oscillations during working memory tasks compared to the infrequent gamers. Despite this electrophysiological difference, both groups demonstrated comparable task performance. The attenuated alpha increase observed in frequent gamers relative to the infrequent gamers may indicate a shift away from transient external attentional suppression toward a more sustained and diffuse attentional mode, as well as enhanced top-down attentional allocation processes potentially influenced by video game play (Hilla et al., 2020; Bediou et al., 2018). Frequent video game play requires attentional control, hand-eye coordination, strategic thinking, and rapid decision-making, all of which constitute cognitively demanding activities associated with a high cognitive load (Gong et al., 2019). It has been suggested that, as a result of frequent video gaming, individuals may reduce cognitive load by employing more automated, task-specific strategies. Additionally, these individuals have been reported to possess enhanced information processing capabilities (Gong et al., 2019). Consistent with this, our findings showing diminished increases in both visual and auditory alpha responses in frequent gamers-despite similar task performance-suggest that frequent gamers may have developed more efficient neural processing strategies by utilizing different cognitive approaches during task execution.

In the present study, we focused on the early post-stimulus time period to investigate increased alpha power upon application of the stimulation. Our results revealed that the infrequent gamer group had higher alpha power in the early post-stimulus period, compared to the frequent gamer group. This increase of the event-related alpha responses in the early time period following stimulus onset can be interpreted as event-related synchronization (ERS), reflecting inhibitory or top-down attentional control processes that suppress irrelevant or distracting inputs (Jensen and Mazaheri, 2010; Pfurtscheller and Da Silva, 1999; Klimesch et al., 2007). Although followed by a global decrease of the alpha power in the later time period, called event-related desynchronization (ERD), ERD was not directly analyzed in the present study. Previous studies have shown that such later alpha decreases correspond to cortical activation and active information processing following the initial inhibitory period (Güntekin et al., 2023; Güntekin et al., 2020; Sato et al., 2018). The pattern observed in our study-early alpha synchronization followed by a later reduction-may represent the temporal dynamics of alpha oscillations, reflecting a transition from inhibitory modulation to cortical engagement. In addition, our results demonstrate regional specificity in alpha modulation in line with previous studies (Tuladhar et al., 2007), with the infrequent gamer group exhibiting higher alpha power over parietal and occipital locations compared to the frequent gamer group. This posterior alpha enhancement may reflect inhibitory control mechanisms and attentional modulation required to suppress task-irrelevant information (Jensen and Mazaheri, 2010; Klimesch et al., 2007). The reduced posterior alpha response in the frequent gamer group may indicate a more efficient or automatized processing strategy, consistent with enhanced attentional allocation due to extensive video game experience. These findings represent that posterior alpha oscillations are physiologically meaningful markers of cognitive control.

### 4.1 Limitations

Several limitations of the present study should be acknowledged. First, concerns the scope of frequency-band analyses. The present study focused on the delta, theta, and alpha frequency bands because delta, theta, and alpha oscillatory responses play crucial roles in attention and memory-related processes (Başar et al., 2001; Güntekin et al., 2022; Güntekin and Başar, 2016; Klimesch, 1999; Sauseng et al., 2010; Klimesch, 2012; Yıldırım et al., 2024). Similarly, higher frequency bands such as beta and gamma are also associated with cognitive functions (Wróbel, 2000; Güntekin et al., 2013; Herrmann et al., 2004; Debener et al., 2003). However, both beta and gamma oscillatory responses have several sub-bands. These sub-bands are associated with distinct cognitive and neural processes (Başar et al., 2015; Başar et al., 2017; Ghaderi et al., 2018); thus, they should be evaluated in more detail. These higher frequency bands, such as beta and gamma, and their subcomponents, may be examined in future studies further to elucidate their potential contributions to memory-related neural dynamics. Second, the relatively small sample size (*N* = 23) limits the generalizability of the findings. Therefore, this research should be considered a pilot study, and future investigations with larger and more diverse samples are needed to validate and extend these preliminary results. Third, although post-hoc corrections (Tukey tests) were applied within each ANOVA, conducting multiple separate ANOVAs for different ERO parameters and tasks may still increase the risk of Type I error. Future studies should implement global correction methods (e.g., FDR or Bonferroni) or multivariate approaches (e.g., MANOVA) to provide stronger control over false positives and enhance the overall statistical rigor and reliability of the findings.

## 5 Conclusion

In conclusion, our findings provide novel evidence that frequent engagement with video games may differentially modulate neural dynamics associated with visual and auditory memory processing. The observed alterations in event-related delta, theta, and alpha oscillations suggest that frequent gamers recruit distinct neurocognitive strategies during memory tasks-strategies that may reflect enhanced efficiency in certain domains (e.g., sensory filtering, lateralized processing), despite similar overt behavioral performance. These neurophysiological differences underscore the potential of video gaming to shape underlying brain mechanisms, particularly in attentional allocation and memory encoding. Importantly, our results open new avenues for exploring how such gaming-induced adaptations might be harnessed in applied contexts such as cognitive training, education, or rehabilitation.

## Data Availability

The raw data supporting the conclusions of this article will be made available by the authors, without undue reservation.
