# Supplementary figures and images for: Frequent video game playing alters low-frequency event-related EEG brain oscillations
Source: Front Psychol. 2025 Nov 19;16:1693697. doi: 10.3389/fpsyg.2025.1693697 (PMC12673345; doi:10.3389/fpsyg.2025.1693697)

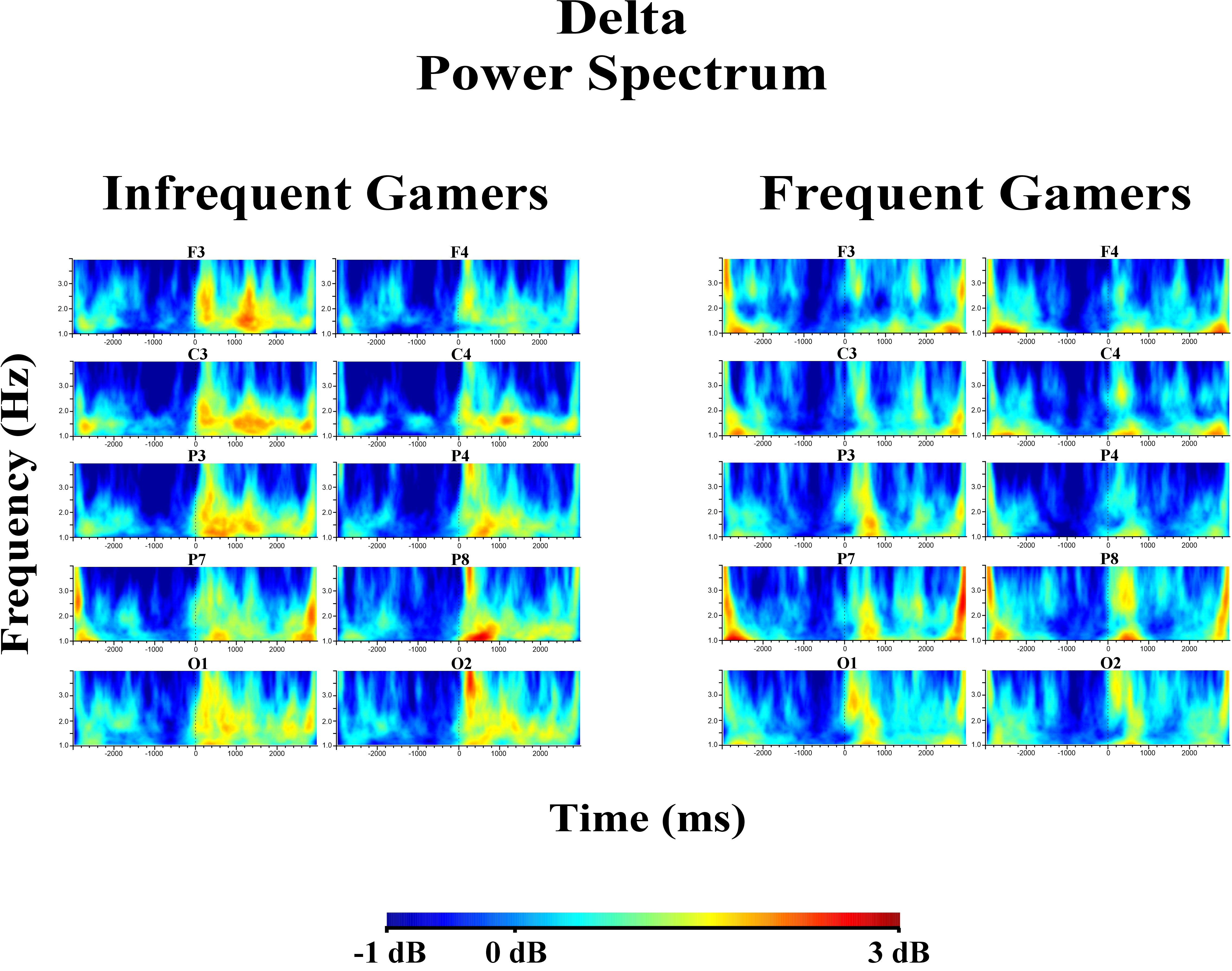

Supplement: Supplementary file 1 [file Image_1.jpeg]

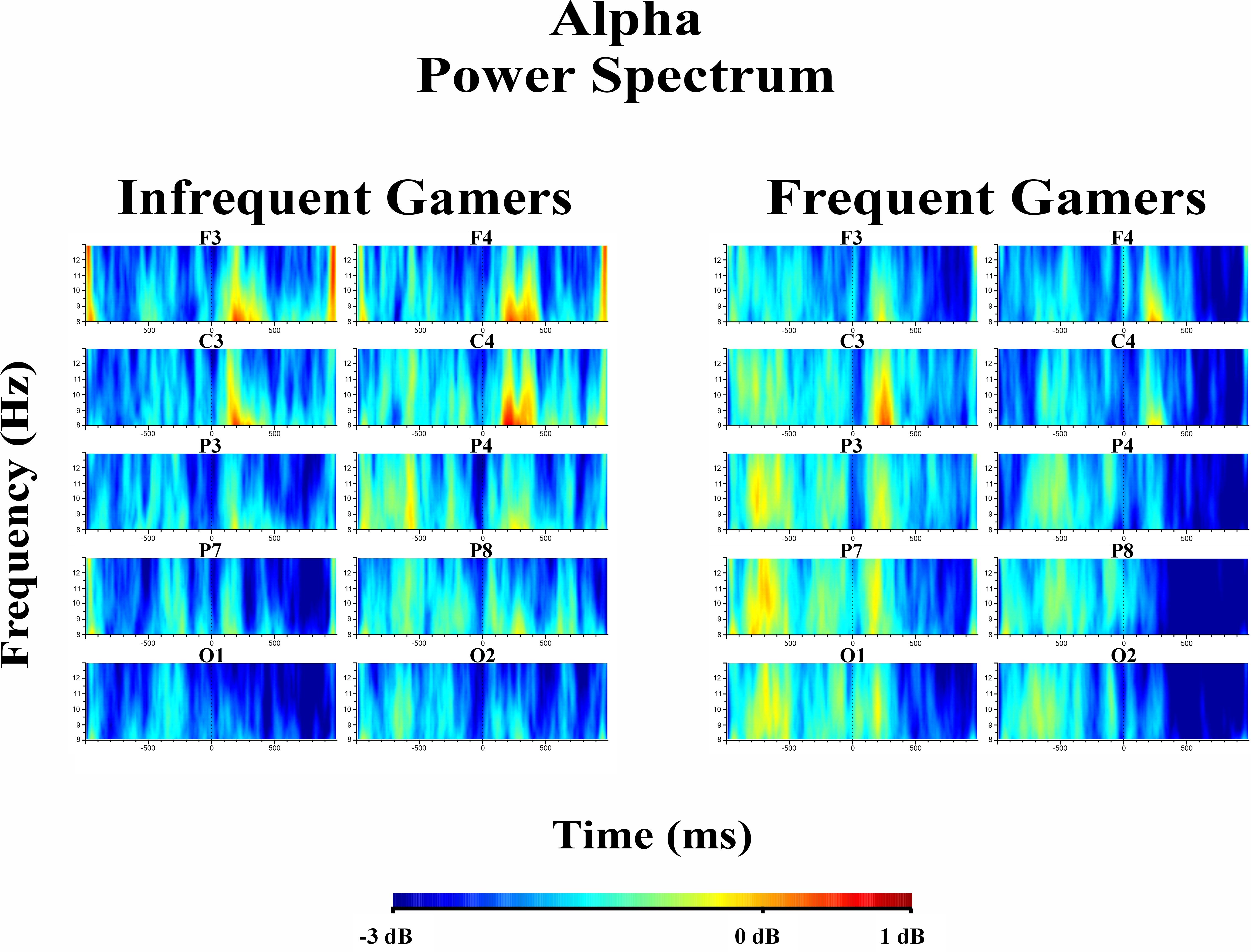

Supplement: Supplementary file 2 [file Image_2.jpeg]

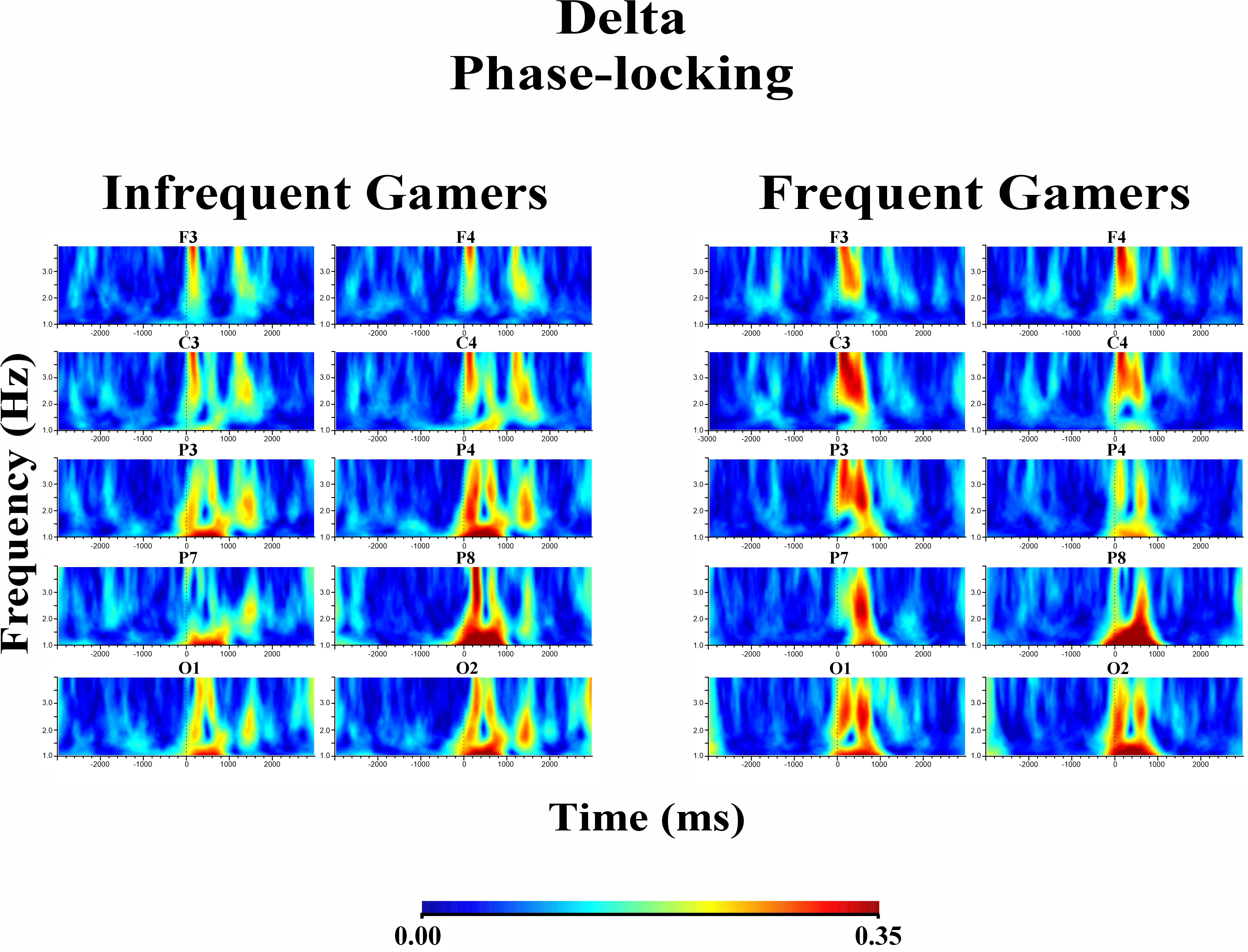

Supplement: Supplementary file 3 [file Image_3.jpeg]

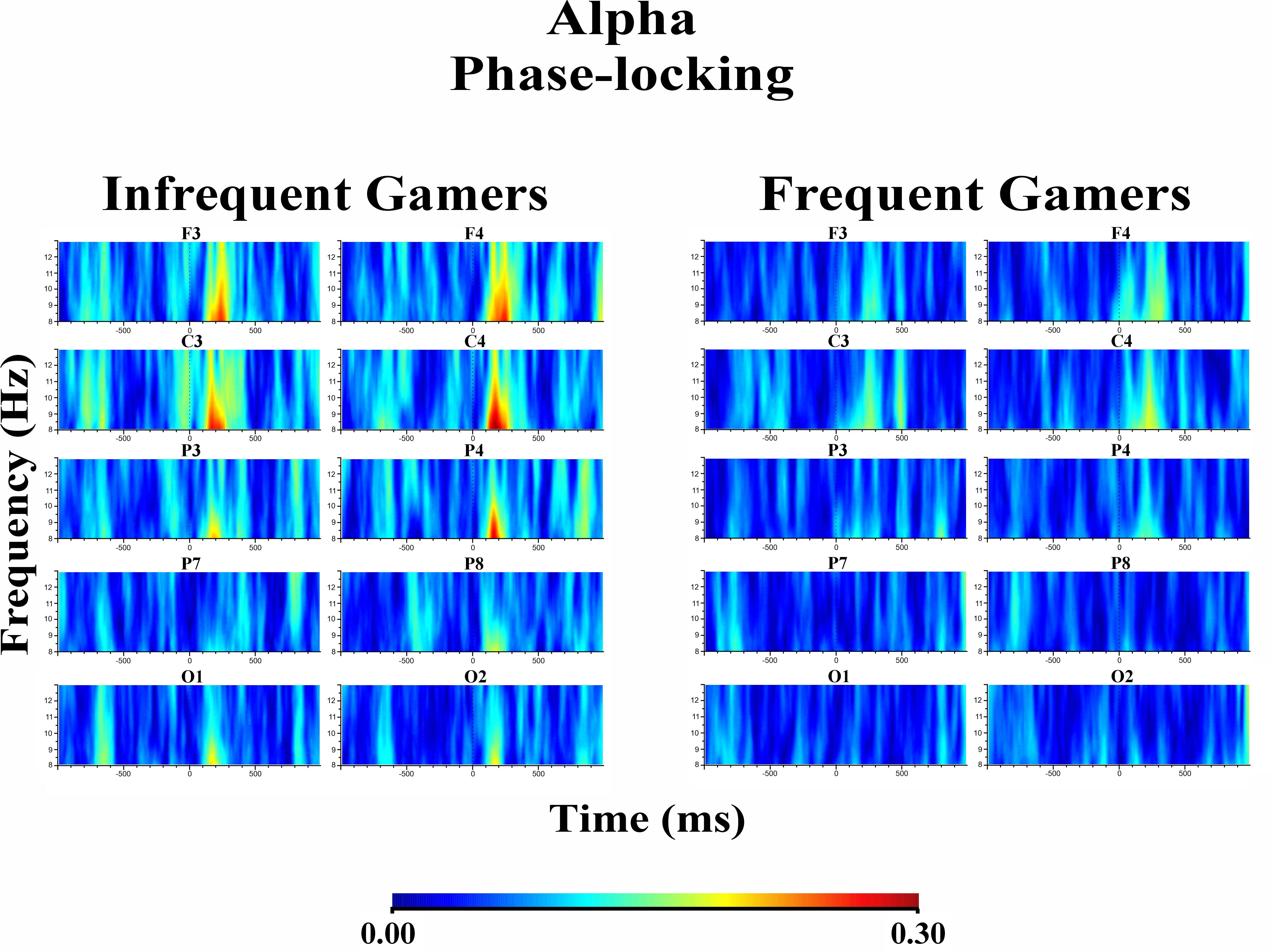

Supplement: Supplementary file 4 [file Image_4.jpeg]
